# Supporting residents’ expression of sexuality: the initial construction of a sexuality assessment tool for residential aged care facilities

**DOI:** 10.1186/1471-2318-14-82

**Published:** 2014-06-30

**Authors:** Michael Bauer, Deirdre Fetherstonhaugh, Laura Tarzia, Rhonda Nay, Elizabeth Beattie

**Affiliations:** 1 Australian Centre for Evidence Based Aged Care; Australian Institute for Primary Care and Ageing, La Trobe University, Melbourne Campus, Victoria 3086, Australia; 2 Dementia Collaborative Research Centre (DCRC)-Carers and Consumers, School of Nursing and Midwifery, Queensland University of Technology, Queensland 4059, Australia

**Keywords:** Sexuality, Tool development, Older people, Residential aged care, Dementia

## Abstract

**Background:**

Sexuality is a key component of quality of life and well-being and a need to express one’s sexuality continues into old age. Staff and families in residential aged care facilities often find expressions of sexuality by residents, particularly those living with dementia, challenging and facilities often struggle to address individuals’ needs in this area. This paper describes the development of an assessment tool which enables residential aged care facilities to identify how supportive their organisation is of all residents’ expression of their sexuality, and thereby improve where required.

**Methods:**

Multi-phase design using qualitative methods and a Delphi technique. Tool items were derived from the literature and verified by qualitative interviews with aged care facility staff, residents and families. The final item pool was confirmed via a reactive Delphi process.

**Results:**

A final item pool of sixty-nine items grouped into seven key areas allows facilities to score their compliance with the areas identified as being supportive of older people’s expression of their sexuality in a residential aged care environment.

**Conclusions:**

The sexuality assessment tool (SexAT) guides practice to support the normalization of sexuality in aged care homes and assists facilities to identify where enhancements to the environment, policies, procedures and practices, information and education/training are required. The tool also enables facilities to monitor initiatives in these areas over time.

## Background

Sexuality is a broad multi-dimensional construct which encompasses relationships, romance, intimacy (ranging from simple touching and hugging, to sexually explicit contact), gender, grooming, dress and styling. Being able to express our sexuality is known to be important to health, well-being, quality of life [1-3] and furthermore, human rights [4]. The desire or need to express one’s sexuality does not expire with age and for many older people including those living in aged care facilities, sexuality continues to be important. A recent study by Bauer et al. [5] found that for many residents in aged care facilities, both with and without dementia, sexuality “still matters”.

The emergence of recent literature tackling the legal, ethical and policy challenges in respect to the expression of older people’s sexuality in care homes (including those with dementia) [6-9] further supports the importance of expression of sexuality as an integral and legitimate need for people living in residential care environments.

However, although there is an increasing emphasis on the application of person-centred approaches to care delivery [6,10-12], many residential aged care service providers still neglect to recognise and address sexuality as a component of wellbeing. Many are unaware of the importance of the multiple facets of the care environment that impact on a resident’s ability to express their sexuality [13-16] and are unaware of what sort of support may be required.

The challenges and difficulties for older people with respect to the expression of their sexuality in the care environment have been repeatedly identified in the literature and include negative and judgemental staff attitudes [17,18]; inadequate knowledge and training [19,20], including around the needs of people who identify as gay, lesbian, bisexual, transgender or intersex (GLBTI) [21,22]; a problem based view of sexuality for people with dementia [23-27]; the prioritisation of other aspects of a resident’s wellbeing over sexuality [28] and a lack of privacy [8]. It is clear that there is scope for aged care facilities to raise their level of awareness and understanding of the needs of older people with respect to sexuality and how to best support its expression [6]. A major barrier to practice change in this area is identifying what resources or other initiatives may be required within an organisation so that it can become more supportive of residents’ expression of their sexuality. Identifying gaps remains difficult, as there are no tools available to appraise a facility’s milieu with respect to meeting residents’ sexuality needs.

This paper describes the development process of a self-report tool for residential aged care facilities. The ‘Sexuality Assessment Tool (SexAT)’ enables aged care providers to assess how well the organisation overall supports the sexuality needs of *all* residents including those with dementia. The tool aims to assist facilities to support the expression of residents’ sexuality by: (1) identifying areas where improvements in the environment, policies, procedures and practices, information provision and education/training may be required and (2); enabling monitoring of these areas over time.

## Methods

The SexAT was developed using a multi-phase design [29]. An initial pool of items was constructed from the literature. Items were confirmed by interviewing facility staff, residents, and residents’ family members and verified by facilities and a Delphi process. This process refined and reduced the initial pool of items and confirmed the content validity of the tool. The project received approval by the La Trobe University human ethics committee (UHEC No: 10–040).

### Initial tool item construction

The initial pool of items for the SexAT was conceptualised by the research team on the basis of a review of the literature (including grey literature) on the issues and obstacles to the expression of sexuality in aged care. To identify any other unreported barriers and enablers which were unique to the Australian residential aged care context, an exploratory qualitative study was carried out. One-on-one interviews or focus groups to explore residential aged care facility staff (all levels of care, allied health, leisure/activity/lifestyle, managers) (*n = 46)*, residents with and without dementia (*n = 5* and *11* respectively) and residents’ family members (*n = 7*) were conducted to explore participants’ views and attitudes towards sexuality in aged care and the perceived needs and barriers to its expression. Written informed consent to participate was obtained from all research participants. Where participants had dementia assent was sought and written consent obtained from their guardian. Six aged care facilities representing public, private and the not-for-profit sectors in two Australian states (Victoria and Queensland) participated in this stage of the tool’s development. Data from the interviews are reported elsewhere [5,30].

### Tool item refinement using a Delphi process

The Delphi technique was developed in the 1940s, and is a structured process designed to gain consensus from a range of experts. Traditionally, it involves a series of rounds, in which participants (or panellists) raise questions or issues on a particular topic. The feedback is summarised and sent back to participants for further review and comment, and this process continues until consensus is reached [31]. Benefits of the Delphi process include: the ability to include individuals from diverse geographic locations and areas of expertise; the opportunity for all panellists to contribute equally to the process without one individual dominating the discussion; anonymity for participants; and the ability to acquire valuable information when there is a lack of existing empirical data on the topic [31,32].

Over time, many different versions of the Delphi method have been developed, with various aspects of the process being modified to suit a particular application. In this case, a ‘reactive’ Delphi process was used, which involves “asking respondents to react to previously prepared information rather than to generate lists of items” [32]. In the context of this study this entailed responding to our draft list of items. A heterogeneous sample of participants was recruited for the Delphi so that a broad spectrum of perspectives and expertise in the topic area were represented. One of the criticisms of the selection of ‘experts’ for a Delphi is that this approach relies on the expertise of individuals, who may not always possess a sufficiently comprehensive knowledge of the area in question [31]. Because the SexAT needed to be able to be operationalised in a residential aged care environment, experts for the Delphi who also had extensive knowledge of this environment were purposely asked to participate. A thirteen member panel of national and international experts from a range of discipline areas including law, ethics, medicine, nursing, sexual health and education, dementia care, facility management and aged care governance reviewed the draft tool.

The Delphi members were asked to review and rank the importance of tool items on a 5-point Likert scale [‘Not at all important’ (1) to ‘Absolutely essential’ (5)]. In addition, critique and comment was sought on the usefulness, appropriateness and clarity of items, item construction and readability, and whether items were comprehensive in their coverage of the issues. The Delphi members were also asked to provide any additional items, reframe items and otherwise edit items to improve the tool.

## Results

The qualitative interview findings confirmed the initial item pool derived from the literature and other documents, as well as raising a number of additional issues such as residents’ access to the services of paid sex workers; the need for support for staff members who were uncomfortable with a resident’s expression of their sexuality; and the lack of availability of double beds within aged care facilities. The initial item pool was sent to two aged care facilities for critical review and comment. Feedback from the facilities identified a number of additional topics around: record keeping and confidentiality of information; the effects of medication; the need for additional staff training and referral; the existence of environmental constraints that impacted on privacy provision; and difficulties dealing with family members.

On the basis of this feedback additional items were constructed and a number of items were re-worded, resulting in an initial item pool of 99 items. Items were grouped into seven sections which reflected the key organisational characteristics which impacted on residents’ rights and ability to express their sexuality: 1) policies and procedures (19 items); 2) assessment and documentation (18 items); 3) staff education, knowledge and attitudes (24 items); 4) resident education and support (16 items); 5) family education and support (8 items); 6) the physical environment (6 items) and 7) safety and risk management (8 items).

The 99 items clustered according to the above sections, were emailed to the Delphi panel for rating. There was a 92% response rate (n = 12) and all but seven items scored an average rating of 4 or more, representing an 80% consensus on the relevance of 92 items for the first iteration of the tool. Five out of the seven items rated less than 4 out of 5 were removed from the tool. The two remaining items related to the provision of sex education for residents and the provision of written information on sexually transmitted diseases and sexual health. These were, after some discussion amongst the research team, left in the item pool for the second Delphi round as they were considered to be too important to discard in view of recent literature highlighting that amongst older people there is low condom use [33] and an increase in sexually transmitted infections [34]. A further 11 items were discarded because they were noted to duplicate other items (n = 6), or because they were considered to be too personal/sensitive and/or, inappropriate (n = 5). Item exclusion and amalgamation reduced the original item pool to 83 items. Prior to the next Delphi round, the non-participating Delphi member was replaced.

The 83 items were again sent out to the Delphi panel who were asked to rate each item’s relevance on the same 5-point Likert scale, in addition to providing qualitative comments. There was a 100% response rate (n = 13). Three items scored an average rating of less than 4 out of 5 for relevance and two of these items were removed; the provision of sex education (retained from round 1), and the provision of written information about sexual expression in large print format for residents with visual impairment. The third item rated less than 4 was reworded from “Assistance is provided to residents who request sexually explicit materials to use in the privacy of their own rooms” to “Residents who wish to exercise their rights to use sex aids will be supported to do so in the privacy of their own rooms”. The rating for the item on the provision of written information on sexually transmitted diseases and sexual health (retained from round 1) increased from 3.8 to 4 and so was retained; however a wording change was made from “diseases” to “infections” to reflect contemporary nomenclature. As in round one, qualitative feedback identified a further 12 items which were removed from the tool because they were repetitive (n = 10) or seen to be inappropriate for people with dementia (n = 2), leaving a final total item pool of 69. A number of other edits were made to the wording of some items for clarity. Given the high level of consensus amongst the expert panel a third Delphi round was not considered necessary.

### Content of the tool

The tool takes the form of a twelve page booklet. The first part of the tool provides a background to the tool’s development and its purpose and a list of definitions to enhance understanding of terms used throughout, such as ‘sexuality’ , ‘sexual assault’ , ‘sexual behaviour’ , ‘staff’ etc. Definitions were included as it was evident to the research team when interviews and focus groups were conducted that people can have a very different understanding of what might be considered commonly understood terms. The second part of the tool explains how the tool is to be used. This is followed by seven sections comprising the tool items (see summary list of final tool items). Some section headings were reworded to more accurately represent the final item pool (Table 1). The final part of the tool gives users information about scoring, with each score category including suggestions for improvement, as well as positive recognition about what the facility might be doing well. A list of helpful resources available on the internet is also provided to assist facilities with quality improvement in this area. The full tool is available for free download at: http://www.dementiaresearch.org.au/images/dcrc/output-files/678-dcrc_formatted_sexat_jan_10_2014.pdf

**Table 1:** Final item pool sections and number of items

1	Facility policies	15
2	Determining the needs of the Older Person	7
3	Staff Education and Training	21
4	Information and Support for Older People	9
5	Information and Support for Families	3
6	The Physical Environment	6
7	Safety and Risk Management	8
Total		69

Summary list of final tool items

Safety and Risk Management

The facility will investigate, act on and prevent behaviours which impinge upon the rights of others or cause others to feel harassed.

Chemical or physical restraint will not be used except in a crisis situation where the risk of harm to residents or staff is present.

The availability of an individualised activity program that is meaningful for residents with dementia who display behaviours that impinge upon the rights of others.

Trained staff assess the ability of a resident with dementia to consent/assent to intimacy on an episode-by-episode basis.

Staff are assessed on their knowledge of current legislation surrounding sexual abuse or reportable assaults.

A risk assessment is performed on residents to determine any safety issues connected with expression of sexuality.

Staff are trained to recognise signs of sexual assault or abuse (past and present).

Staff are trained to recognise signs of unwanted sexual contact.

The Physical Environment

Provision of private spaces.

Provision of opportunities to express sexuality in a social setting.

Residents are able to request sexually explicit materials to use in the privacy of their own rooms.

Availability of double or adjoining rooms for residents who wish to live as a couple.

Availability of double beds.

Availability of privacy measures for individuals who are sharing a room but are not a couple.

Information and Support for Families

Education on older adults’ sexuality and rights.

Availability of identified and trained staff to support families.

Availability of written information on sexuality (in a format family can understand).

Information and Support for Older People

Availability of trained staff to discuss sexuality and provide support.

Information is provided to residents about the above trained staff.

Availability of information for residents in a format they can understand on (5 items): sexual health; consent; assault; sexual orientation/identity and discrimination and rights.

If requested availability of information for residents on sexual aids/lubricants/condoms and audio-visual aids.

Information on who to approach if perceived abuse and/or discrimination.

Staff Education and Training

Provision of education for varying levels of staff on (10 items): sexuality and ageing; personhood; dementia; sexual health; risk management; managing conflict (families and residents); sexual discrimination; consent and decision-making; privacy and medications.

Competencies with respect to staff knowledge of policies and procedures around sexuality.

For senior staff/management: dealing with issues of concern raised by staff.

Guidelines on what is appropriate/inappropriate sexual expression.

Differentiating healthy sexuality and behaviours of other unmet needs.

Guidelines about appropriate and inappropriate levels of assistance staff can offer residents.

Communication skills for staff to assist them to respond to residents and families.

The availability of written information on sexuality for staff.

The provision of summaries of relevant legislation relating to privacy, guardianship and residents’ rights.

Appraisal of staff attitudes towards sexuality before and after education.

Competencies for staff qualified to have conversations about sexuality and collect information.

Competencies to evaluate staff performance in respecting residents’ right.

Determining the needs of the older person

Use of an assessment tool by trained staff to identify residents’ needs.

Behaviours that impinge on the rights of others are documented and investigated.

Residents are given the opportunity to discuss effects of medications.

Residents are given the opportunity to raise and discuss facility support for the expression of their sexuality and anything that may be impacting on it with appropriately trained staff.

Residents are asked about and given the opportunity to discuss their personal presentation and styling.

Residents are asked if they are satisfied with their opportunities to socialise.

Promotional/marketing materials reflect facility support for residents’ rights to express their sexualities.

Facility Policies

Recognition and support of residents’ right to express sexualities (providing it does not impinge upon other’s rights).

Recognition of right to privacy

Recognition of a resident’s right to use aids/equipment/visuals in their room.

Incorporation of sexuality into assessment and care planning.

Residents are given the opportunity to discuss their needs with appropriately trained staff.

Confidentiality of information where there is no cognitive impairment.

Provision of ‘Do Not Disturb’ signage for doors.

Knock and wait for permission to enter requirement before entering rooms (unless an emergency).

Adherence to ‘Do Not Disturb’ signage (unless an emergency).

Unacceptability of discriminatory/sexist/ageist/homophobic language or behaviour in the facility.

Staff offer same level of assistance with personal/intimate hygiene care surrounding sexual activity as is given for other activities of daily living such as toileting.

Resident’s right to access the services of a sex worker (if legal).

Support and assistance for family to understand residents’ rights where the person has a cognitive impairment and where there is a conflict.

Support for staff who feel uncomfortable about a resident’s sexual expression.

Support for family members who feel uncomfortable about a resident’s sexual expression.

### Using the tool

The SexAT is designed to be self-administered by an aged care facility manager, or other senior staff member who knows the facility well and is familiar with the policies, procedures, and strategies currently in place. For simplicity of use a ‘Yes’ , ‘No’ , or ‘Sometimes’ format was adopted with responses tallied at the end of each section; giving the facility a score for each section, as well as an overall score. This allows facilities to assess their compliance in individual areas and overall.

## Discussion

There is a recognised role for assessment tools in improving care for older people living in aged care facilities. Rheume and Mitty [35] have noted that staff working in aged care facilities “need…tools in order to address residents’ [sexual expression] and the many barriers to intimacy” (p.342). The development of the SexAT is a step towards the normalisation of sexuality for older people (including those with dementia) living in residential aged care facilities as it identifies a range of factors that impact on its expression, and enables facilities to target areas of improvement. It sets up the expectation that facilities should recognise sexuality as a meaningful and legitimate component of care. The literature continually highlights aspects of the facility environment which are barriers to the expression of sexuality by older people with and without dementia. The SexAT encourages facilities to critically examine their physical spaces as well as policies, practices and information/training needs in order to create and promote an environment that is more conducive and supportive of the needs of older people as opposed to the convenience of the staff.

Although, in Australia, the Charter of Residents’ Rights and Responsibilities [36] stipulates the right of residents to privacy and control over their relationships, there is no definitive guidance for aged care facilities with respect to how a facility can be made more conducive to the expression of sexuality. Use of the tool will allow facilities to identify and approach issues in a structured and individualised manner by setting up comprehensive ‘standards’ that facilities can aim to meet in order to be more supportive of residents’ needs.

It could be argued that treating sexuality as a separate issue perpetuates its taboo status, and that a person-centred approach to care should encompass sexuality. We believe however that the centrality of the expression of sexuality to health, wellbeing and quality of life, as well as its history of neglect as a component of older people’s healthcare needs, justifies the use of a dedicated tool such as this. As Wallace [37] has noted, it is rare for care plans to address the sexuality needs of residents, despite the potential benefits to person-centred care.

## Conclusion

Currently no tools exist in the literature to assess residential aged care facilities’ support of their residents’ sexuality. The SexAT is an easy to use tool that fills a gap in aged care service delivery. It provides a framework for aged care organisations to identify how well their environment and practices recognise and support the rights of older people to express their sexuality, including for people with dementia. By auditing against best practice, aged care services can ascertain areas in which they need to improve and develop and implement strategies to do so. Future work will focus on knowledge translation and the utility of the tool in practice.

## Competing interests

The authors declare that they have no competing interests.

## Authors’ contribution

MB, RN, DF and EB were responsible for the study conception and design. MB, DF and LT acquired and analysed the data. MB and LT drafted the manuscript and DF, RN and EB contributed to refinement. All authors read and approved the final manuscript.

## Pre-publication history

The pre-publication history for this paper can be accessed here:

http://www.biomedcentral.com/1471-2318/14/82/prepub
